# An 11bp Region with Stem Formation Potential Is Essential for *de novo* DNA Methylation of the RPS Element

**DOI:** 10.1371/journal.pone.0063652

**Published:** 2013-05-06

**Authors:** Matthew Gentry, Peter Meyer

**Affiliations:** Center for Plant Sciences, University of Leeds, Leeds, United Kingdom; St Jude Children's Research Hospital, United States of America

## Abstract

The initiation of DNA methylation in *Arabidopsis* is controlled by the RNA-directed DNA methylation (RdDM) pathway that uses 24nt siRNAs to recruit *de novo* methyltransferase DRM2 to the target site. We previously described the *REPETITIVE PETUNIA SEQUENCE* (RPS) fragment that acts as a hot spot for *de novo* methylation, for which it requires the cooperative activity of all three methyltransferases MET1, CMT3 and DRM2, but not the RdDM pathway. RPS contains two identical 11nt elements in inverted orientation, interrupted by a 18nt spacer, which resembles the features of a stemloop structure. The analysis of deletion/substitution derivatives of this region showed that deletion of one 11nt element RPS is sufficient to eliminate *de novo* methylation of RPS. In addition, deletion of a 10nt region directly adjacent to one of the 11nt elements, significantly reduced *de novo* methylation. When both 11nt regions were replaced by two 11nt elements with altered DNA sequence but unchanged inverted repeat homology, DNA methylation was not affected, indicating that *de novo* methylation was not targeted to a specific DNA sequence element. These data suggest that *de novo* DNA methylation is attracted by a secondary structure to which the two 11nt elements contribute, and that the adjacent 10nt region influences the stability of this structure. This resembles the recognition of structural features by DNA methyltransferases in animals and suggests that similar mechanisms exist in plants.

## Introduction

Cytosine methylation is an ancient modification system that has diversified into different biological roles including restriction modification systems in bacteria, and epigenetic regulation of gene expression and genome structure in most eukaryotes, where cytosine methylation works in combination with histone modifications [1]. Research on the control and biological effects of 5 methyl cytosine (5mC) has focussed on understanding how 5mC patterns are maintained and removed, and especially how defined genomic regions are selected as DNA methylation targets. DNA methylation in *Arabidopsis* is established at cytosine in symmetrical (CG, CNG) and non-symmetrical (CNN) sequence context. All three methylation types are detected in pericentromeric heterochromatin, transposons and other repetitive sequences, which are associated with _∼_24nt small interfering RNAs, while about one third of expressed genes contain predominantly CG methylation marks in body regions, for which no matching small RNAs are detected [2]; [3]. This distribution conforms to the model that DNA methylation is established via the RNA-directed DNA methylation (RdDM) pathway, which uses small RNAs that associate with ARGONAUTE AGO4 [4] or AGO6 [5] complexes to guide *de novo* DNA methyltransferase DOMAINS REARRANGED METHYLTRANSFERASE 2 (DRM2) to homologous regions [6]; [7]. The small RNAs derive from double-stranded RNAs, which are cleaved by the DICER-LIKE3 (DCL3) endonuclease. Double stranded transcripts could derive from inverted repeats, from antagonistically transcribed regions, or from reverse transcription of ssRNA by RNA-DEPENDENT RNA POLYMERASE 2 (RDR2).

In the filamentous fungus *Neurospora crassa*, which also contains methylated cytosines in all sequence contexts, DNA methylation does not depend on small RNA signals [8] but on histone marks. Methylation is mediated by cooperative recognition of the minor groove of multiple short A:T tracts that derive from a repeat-induced point mutation (RIP) mechanism that induces C to T transition mutations at duplicate sequences [9]. This leads to the establishment of H3K9 trimethylation marks by H3K9 methyltransferase DEFECTIVE IN METHYLATION-5 (DIM-5) at RIP regions, which attracts DNA methyltransferase DIM-2 [10]. Plants and animals contain multiple examples for an interplay between histone and DNA modification systems, especially in heterochromatin formation [11]. Mechanisms for initiating heterochromatin formation include siRNA mediated targeting of histone methyltransferases [12]; [13], but also RNA independent targeting of sequence-specific DNA-binding proteins that recruit histone modifying enzymes to distinct regions [14]; [15].

The RPS from *Petunia hybrida* is a 1.6 kb fragment that becomes efficiently methylated upon integration into the genome of other species, including the Arabidopsis genome, which does not contain any RPS homologues. RPS contains a number of inverted repeat elements, and *de novo* methylation levels are especially high around a *Hha*I site located within a putative stemloop region [16]. RPS-specific DNA methylation differs from DNA methylation at heterochromatic regions as it does not involve the recognition of sequence repeats nor does it depend on the chromatin-remodelling ATPase DDM1 [17], which is required for H3K9 methylation and DNA methylation in heterochromatic regions [18]. RPS methylation is also independent of DCL3 and RDR2, but it requires the presence of all three DNA Methyltransferases MET1, CMT3 and DRM2. In wildtype, RPS-specific methylation is associated with homologous small RNAs, which are, however, absent in a RDR2 mutant where RPS methylation is reduced but still present. This suggested that RPS initiates DNA methylation via an RdRM-independent mechanism, and that methylation, once it has been established, is recognised and augmented by RdRM pathway functions [17]. To investigate the nature of the signal that initiates RPS methylation, we tested if DNA methylation was affected by deletions or substitutions within and around the putative stemloop region. We find that removal of one of the 11nt inverted repeat elements is sufficient to eliminate RPS methylation, while sequence changes within the 11nt repeats that do not alter pairing homology don't affect DNA methylation efficiency, which suggests that RPS-specific methylation is initiated via the recognition of structural features rather than specific sequence elements.

## Results

**Figure 1:**
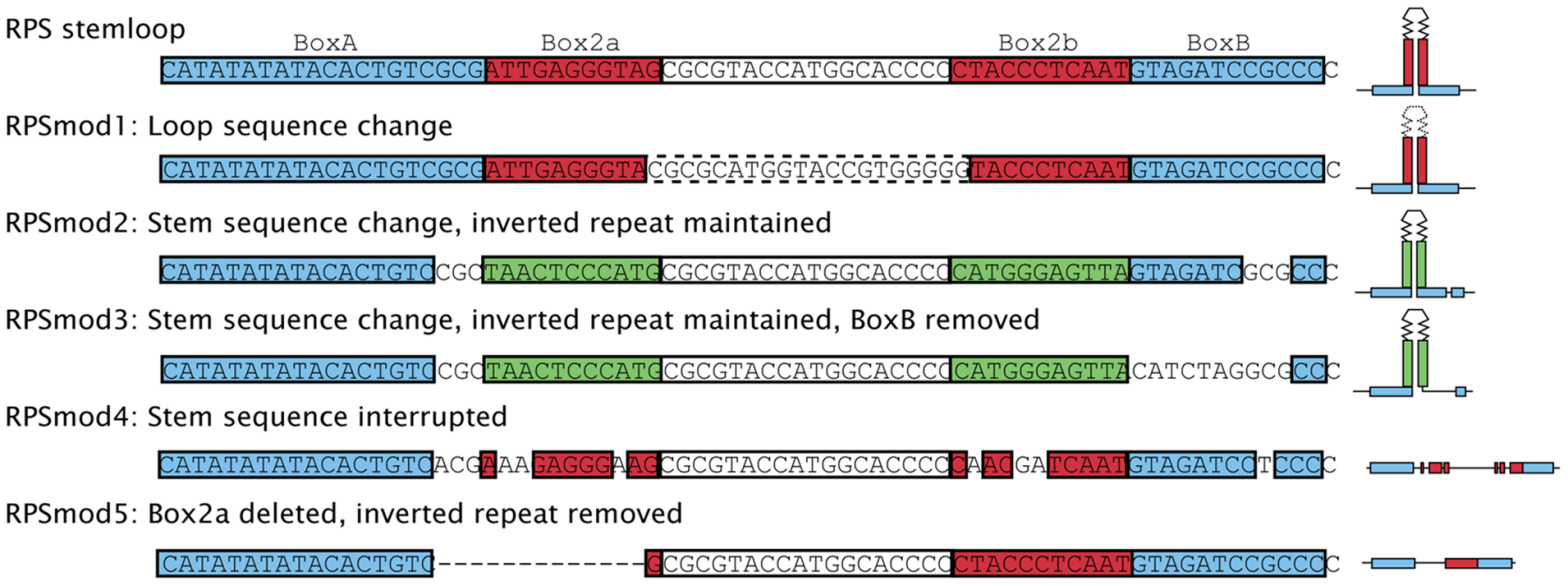
Modifications to the RPS stemloop. Unmodified RPSstemloop is shown at the top along with coloured regions for boxA and boxB (blue), and palindromic regions Box2a and Box2b (red). Modifications are indicated by: dashed region in *RPSmod1* indicating change to loop sequence; green boxes in mod2 and mod3 indicate sequence changes that remain palindromic; modification of sequences in *RPSmod2* and *RPSmod3* (boxB), and also *RPSmod4* (box2a and box2b) indicated by missing sections; and deletion of box2a in *RPSmod5* indicated by dashed line. A schematic representation of the putative stem structure predicted by mfold (http://mfold.rna.albany.edu) is given to the right where applicable.

We selected the potential stemloop region between position 559–598 of the RPS fragment [16] as a target for a deletion/substitution analysis, and designed five modification constructs *RPSmod1-5* (Figure 1). In *RPSmod1*, the sequence of the loop region between the 11nt Box2a and 2b stem elements was altered. In *RPSmod2* and *RPSmod3*, DNA sequences of the 11 bp Box2a and 2b stem elements were modified while nucleotide composition and stem formation potential remained unchanged. In addition, 10 nucleotides of BoxB were deleted in *RPSmod3*. In *RPSmod4*, stem formation was weakened by nucleotide substitutions in the 11bp Box2a and 2b stem elements, and in *RPSmod5*, one of the 11nt Box2a stem elements was deleted.

**Figure 2:**
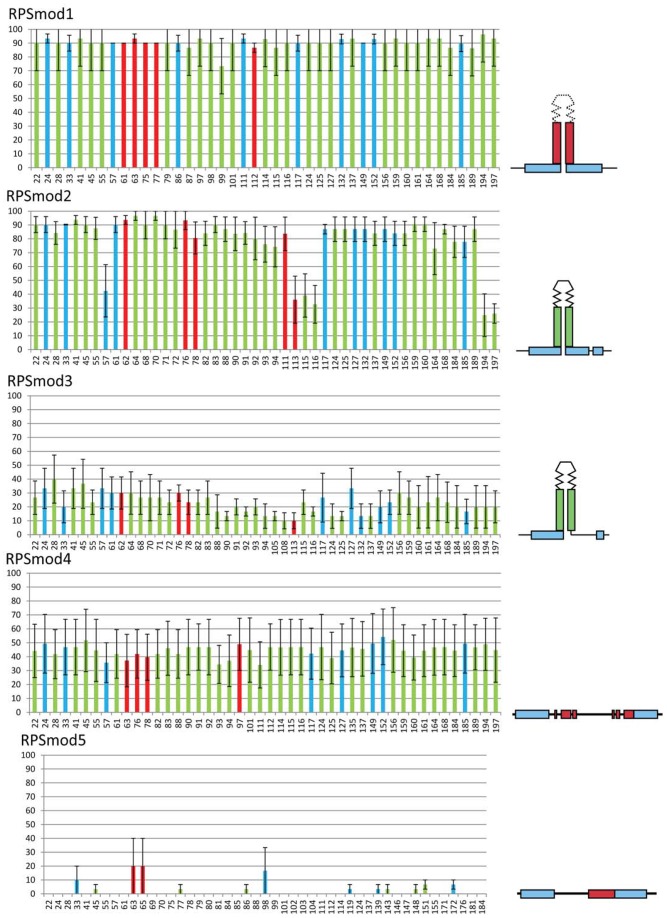
Bisulfite analysis of *RPSmod* lines in a wild-type background. Bars show the percentage of DNA methylation at each cytosine residue indicated on the horizontal axis, with CG methylation in red, CNG methylation in blue and CNN methylation in green. The RPS stemloop region starts at position 64. Error bars represent variation between three single copy lines.

We selected three single copy *Arabidopsis thaliana* transformants for each construct, for which we determined the DNA methylation levels within a 200 bp region around the stemloop region or its modifications (Figure 2). Sequence changes to the loop region in *RPSmod1* transformants had no inhibitory effect on RPS methylation, which reached very high levels both at CG and non-CG targets. Sequence modifications to the Box2 stem region in *RPSmod2* lines, which do not alter stem formation, had only a very minor effect in reducing DNA methylation, which was, however, significantly enhanced when coupled with a 10nt replacement of BoxB in *RPSmod3* transformants. Resolution of the stem structure in *RPSmod4* lowers but does not eliminate methylation, but deletion of one of the 11nt stem elements in *RPSmod5* abolishes methylation.

**Figure 3:**
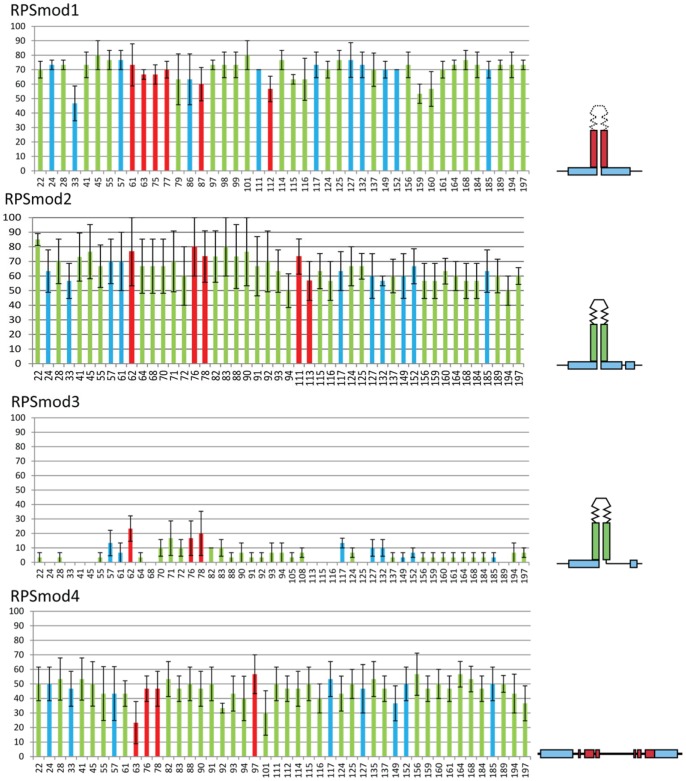
Bisulfite analysis of *RPSmod* lines in a *rdr2* background. CG, CNG and CNN methylation is indicated by red, blue and green bars, respectively. All modifications except *RPSmod4* show a reduction in methylation compared to wild-type lines indicating an amplification role of RDR2 in RPS methylation. Methylation in R*PSmod3* is almost eliminated. *RPSmod4* methylation remains largely unchanged from wild-type.

To investigate if the RdDM pathyway contributed to the efficiency of methylation for those RPS derivatives that still attracted DNA methylation, we repeated the methylation analysis of three single copy transformants in a *rdr2* mutant background (Figure 3). All modifications except *RPSmod4* show a reduction in methylation compared to wild-type lines indicating an amplification role of *RDR2* in RPS methylation. Only *RPSmod4* methylation patterns remain largely unaffected by the absence of RDR2.

The loss of *de novo* methylation in *RPSmod5* suggests that the putative stem region is crucial and possibly represents the initial target region for *de novo* methylation. The methylation analysis of RPS derivatives in *rdr2* confirms our previous model that initial methylation is independent of RdRM functions but that it can be enhanced by the RdRM pathway leading to increased methylation levels and possibly also to spreading of methylation from the initial *de novo* methylation region.

**Figure 4:**
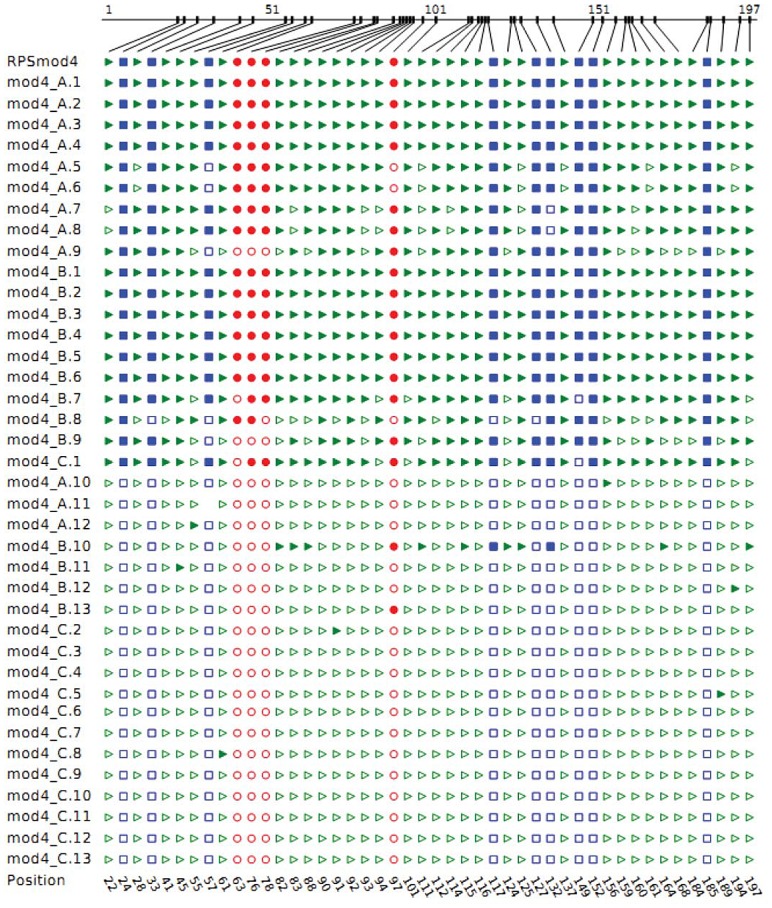
An illustration of the methylation states of individual clones of three *RPSmod4* transformants, lines A, B and C. Methylated sites are marked by filled in for CG sites (red circles), CNG sites (blue squares) and CNN sites (green triangles). Most clones are either >80% methylated or <10% methylated.

**Figure 5:**
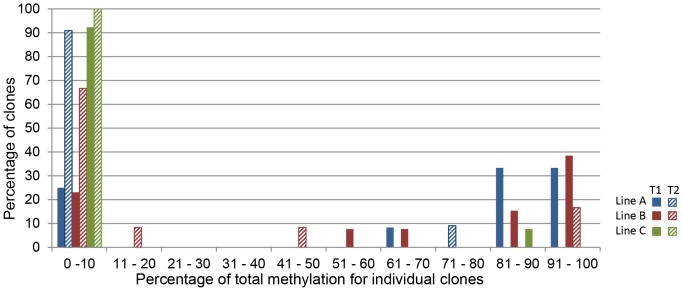
Analysis of hypermethylation rates for *RPSmod4* clones in the next generation. Classification of *RPSmod4* clones according to percentage of total methylation for each independent line. T1 generations (solid bars) show a distinct bimodal distribution of <10% and >80% methylation. For line A, the majority of clones in the T1 generation are highly methylated. Line B shows fewer highly methylated clones and line C shows mostly unmethylated clones. All lines show an increase in unmethylated clones in the T2 generation (striped bars). Line A has no clones showing >80% methylation. Line B shows 2 out of 12 clones still having high levels of methylation and 2 clones with intermediate levels. For line C, no clones showed significant methylation in the T2 generation.

The large error bars associated with methylation patterns of *RPSmod4* transformants highlight that a standard deviation analysis is deceptive as *RPSmod4* lines actually show a bimodal methylation profile (Figure 4). Individual *RPSmod4* clones are either methylated at levels >80% or almost completely unmethylated. These data are in accordance with a ‘switch’ mechanism that alternates between almost complete hypomethylation and hypermethylation of *RPSmod4*, respectively. Individual transformants showed different ratios of almost fully methyated and fully unmethylated clones (Figure 5). To investigate if these observed differences in the hypermethylation frequency represented locus-specific effects, we analysed methylation ratios in the F1 generation of three lines (Figure 5). Our results show that the frequencies at which individual RPSmod4 transgenes become hypermethylated or remain hypomethylated, are not line-specific but appear to be stochastic events.

## Discussion

The RPS is an unusual DNA methylation target as its *de novo* methylation occurs independently of RdRM pathway functions, which only contribute to the augmentation of DNA methylation marks once they have been established [17]. This leaves the question of which regions and features within RPS are responsible for the initiation of DNA methylation. Our data identify the 11nt stem structure as a critical region for the recognition of RPS as a *de novo* methylation target, as DNA methylation is reduced to background levels in *RPSmod5* transformants, which contain a RPS transgene from which one of the 11nt stem segments has been removed. For *RPSmod2* transgenes, where both 11nt stem elements have been replaced with DNA elements with altered sequence but unchanged inverted repeat homology, no significant reduction in DNA methylation efficiency was detected, which supports the assumption that structural features rather than DNA sequence elements guide RPS methylation. While the 11nt region is required for RPS methylation, it is not sufficient, as maintenance of efficient *de novo* methylation is influenced by modifications to the adjacent BoxB region in the *RPSmod3* constructs. This implies that BoxB contributes to the efficiency of secondary structure formation that attracts *de novo* methylation.

Conformational changes of DNA regions are powerful recognition signals for regulatory proteins [19]; [20]; [21] and can also significantly affect target selection of DNA methylation functions [22]. Sequence specificity of mammalian Dnmt1 is altered by supercoiling which might induce alternative secondary structures that affect their interaction with DNA methyltransferases or auxiliary nuclear proteins [23]. Structural features have also been proposed to play a role in the specific recognition of transposition and viral integration intermediates by DNA methylation systems that inactivate invasive DNA [24]. Unusual non-B structures at the highly polymorphic human VNTR region have been proposed to function as a methylation hotspot [25].

In *RPSmod4* transformants, we detect a very unusual DNA methylation profile as individuals clones are either almost completely hypermethylated or hypomethylated. Such a bimodal on-off scenario is in accordance with a conformational switch between an unstructured conformation and a distinct secondary structure that acts as a strong signal for *de novo* methylation. It is therefore conceivable that, while this secondary structure is formed with high efficiency in *RPSmod1*, the sequence replacement in *RPSmod4* has reduced the probability that an appropriate secondary structure is formed. This would generate two classes of molecules, those that don't form the appropriate structure and remain hypomethylated, and those that do form a structure that attracts *de novo* methylation and that become efficiently methylated. It is also conceivable that local *de novo* methylation supports a conformational change that provides an efficient DNA methylation target, and that, once methylation has been initiated, the methylation signal efficiently spreads to neighbouring regions. This is reminiscent of conformational switches observed in cruciforms creating two distinct conformational states with differed affinities for interacting factors [26].

The reduced methylation levels of *RPSmod1-3* in a *rdr2* mutant confirm previous observations about an enhancing influence of RDR2 in RPS methylation and a model that RPS initiates DNA methylation, which serves as a signal to attract RdDM pathway functions that amplify the methylation signal [17]. Interestingly, *RPSmod4* methylation patterns are the only ones that are not significantly augmented by RDR2, which could be explained by the special bimodal distribution of *RPSmod4* transgenes, which are either unmethylated and therefore probably no target for a methylation enhancement mechanism, or which are already methylated at levels that are too high to be significantly enhanced by RDR2 activity.

**Figure 6:**
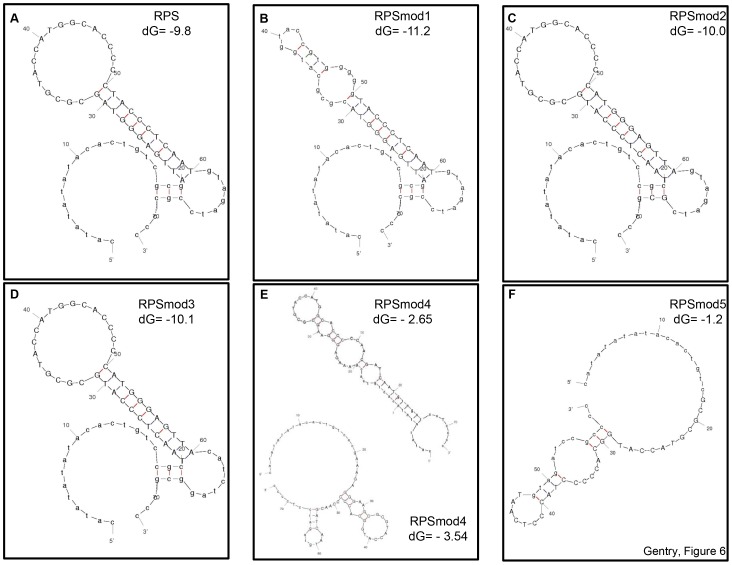
Folding models for RPS derivatives. RPS (A) and the five RPSmod sequences (B–F) were submitted to the DNAfold programme [27] to calculate potential secondary structures and their energy levels. For RPSmod4 (E) two alternative folding options are shown.

In conclusion, our work has identified a critical target element for *de novo* methylation within a putative stem structure, it has shown that functionality of the stem element is not dependent on a specific DNA sequence, and that methylation efficiency is significantly influenced by a small region directly adjacent to the stem region. We don't know the nature of the structural signals responsible for the *de novo* methylation of RPS but we notice that the efficiency of attracting *de novo* methylation among the different RPS modifications correlates with the probability of stemloop formation (Figure 6). For *RPSmod4*, the programme calculates two folding options with strong and weak hairpin potential (Figure 6E), which would be in agreement with a conformational switch between structures that favour hypermethylation and hypomethylation, respectively. It will be interesting to investigate if and which endogenous loci provide structural signals that can establish novel DNA methylation patterns, independent of RdRM pathway functions, and if these regions can undergo conformational changes that makes them as efficient targets for *de novo* DNA methylation similar to some of the RPS variants we examined.

## Experimental Procedures

DNA extraction was performed using a modified CTAB method. 1–2 g of tissue was ground in liquid nitrogen and then transferred to a 2 ml eppendorf tube. 560 µl Extraction buffer (2M NaCl; 20 mM soduim meta-bisulfite; 200 mM TrisHCl, pH 8.0; 70 mM EDTA) was added and the sample was vortexed briefly before adding 180 µl 5% sarcosyl. Samples were incubated at 65°C for 2 hours. Samples were phenol:chloroform extracted and DNA precipitated with ½ volume isopropanol and ½ volume high salt buffer (0.8M Sodium citrate; 1.2M NaCl). The resulting pellet was dissolved in 400 µl TE buffer (pH 8.0), and after addition of RNase (25 µg) was incubated at 65°C for 15 minutes. 400 µl of CTAB buffer (50 mM EDTA; 200 mM TrisHCl, pH 8.0; 125 mM NaCl; 0.04% w/v PVP; 5% β-mercaptoethanol) was added and samples were incubated at 65°C for a further 15 minutes. Samples then underwent two rounds of phenol:chloroform:IAA extraction followed by a further chloroform:IAA (24∶1) clean up. Samples were precipitated with isopropanol.

### RPSmod plasmids design and transformation

Oligonucleotides containing the RPS stemloop modification sequences flanked by SnaBI and BsrGI restriction sites were ordered from MWG biotech. Formation of secondary structures of the modified regions was tested by importing modified regions into DNAfold [27]. To generate the *RPShyg* vector, pGreen0179 [28] was digested with HindIII (New Endgland Biolabs www.neb.com) and ligated with a 1.6 kb RPS HindIII fragment isolated from p35SGUS/RPS [16]. To produce the RPShygdel recipient vector, RPShyg was digested with SnaBI and BsrGI (New England Biolabs www.neb.com) to remove a 232 bp region containing the RPS stemloop. The vector was gel purified using Q-spin Gel Extraction kit (Geneflow, www.geneflow.co.uk) to produce the *RPShygdel* recipient vector. The pre-ordered oligonucleotides were cloned into the pGEM TA cloning vector (Promega, www.promega.com) according to the manufacturer's instructions. Each modified RPS stem loop region was cut out of purified plasmids using SnaBI and BsrGI. Inserts were cloned into *RPShygdel* to generate each *RPSmod* construct. These were cloned into *Agrobacterium tumefaciens* (GV1301) for transformation in *Arabidopsis thaliana* lines by floral dip [29]. Prior to plant transformation, plasmids purified from *Agrobacterium* were sequenced (GATC, www.gatc.com) to ensure no sequence changes had occurred.

### Bisulfite sequencing

Bisulfite sequencing was performed using QIAGEN Epitect conversion kit (www.qiagen.com) according to manufacturer's instructions except the cycling conditions were increased to 90°C 5 min, 60°C 25 min, 90°C 5 min, 60°C 45 min, 90°C 5 min, 60°C 55 min, 90°C 5 min, 60°C 60 min, 90°C 5 min, 60°C 60 min. The RPS region (top strand) was amplified from treated DNA using primers RPStop-F (CTg/aTATTTTTCTCCCTTCA) AND RPStop-R (AAGTAGAAAGGAAAGAGAAAAGGGG). PCR was carried out using myTaq polymerase (Bioline www.bioline.com) under the following conditions: 94°C, 15 secs; 54°C, 20 secs; 72°C 15 secs for 45 cycles generating either a 216 bp (*RPSmod1-4*) or 203 bp (*RPSmod5*) fragment. Amplicons were separated on a 2% agarose gel and excised using using Q-spin Gel Extraction kit (Geneflow, www.geneflow.co.uk). Purified fragments were cloned using the pGEM TA cloning vector (Promega, www.promega.com) according to the manufacturer's instructions and transformed into *E.coli* DH5α competent cells. A minimum of ten bisulfite treated clones were sequenced for each line (GATC, www.gatc.com) and analysed by exporting their sequence to BioEdit [30]. Aligned sequences were sent to Cymate [31] to calculate and illustrate DNA methylation frequencies.
